# C.P titanium/Ti–6Al–4V joint by spark plasma welding: Microstructure and mechanical properties

**DOI:** 10.1016/j.heliyon.2024.e27514

**Published:** 2024-03-03

**Authors:** Saeed Zahabi, Ehsan Mohammad Sharifi, Mehdi Naderi, Mazaher Ramezani, Hossein Jamali, Mohammad Reza Loghman Esatrki

**Affiliations:** Department of Materials Engineering, Malek Ashtar University of Technology, Isfahan, Iran

**Keywords:** Diffusion bond, Spark plasma welding, Dissimilar welding, Ti–6Al–4V, C·P Ti

## Abstract

The welding ability of Ti–6Al–4V alloy is weak due to their two-phase microstructure. On the other hand, friction welding methods lead to significant microstructural changes. In this research, for the first time, pure titanium was successfully joined to the Ti–6Al–4V alloy, without any change in the microstructure and mechanical properties of both alloys, by applying the SPW method. Further, the effects of temperature, pressure, and time of the SPW process on the microstructure and mechanical properties of commercial pure (C.P) titanium joined to the Ti–6Al–4V alloy were investigated. The results indicated that the effect of temperature and pressure on the SPW process was greater than that of time. Further, mechanical properties investigations showed that the yield strength of the joint interface was larger than that of the substrate metal, following which necking and fracture occurred in the pure titanium substrate metal. The alloy (Ti–Ti64) bonded at 800 °C, with a time of 10 min and pressure of 20 MPa, exhibited the superior bonding of 7–9 μm interface thickness, and excellent tensile strength (534 ± 13 MPa) and Vickers micro-hardness (190 ± 5 HV_0.1_). Investigation of the effect of pressure (normal stress) also showed that with an increase in pressure, because of the reduction of the chemical potential of diffusing species, the joint temperature would drop, and the joint could be created at a temperature below 800 °C.

## 1 Introduction

Titanium and its alloys are widely used in the aeronautics industry because of its high strength to density, good corrosion resistance, and high flexibility [[1], [2], [3], [4], [5]]. Rivets aircraft can be regarded as one of the most important applications in which titanium is joined to the Ti–6Al–4V alloy. In some uses such as aeroplane rivets, high flexibility is required on the one side and high strength on the other, while pure titanium alone does not possess all of these properties. Therefore, the Ti–6Al–4V alloy is used because of its suitable strengths, whereas pure titanium is applied to provide high corrosion resistance and flexibility. All sets of components in this alloy provide the required properties of rivets; as such, they should be welded to each other in some way. Further, welding dissimilar metals with suitable mechanical properties is one of the challenges in the industry.

The most common method for welding rivets is frictional welding. However, in this method, because of high welding temperature, unwanted intermetallic phases may be formed, which could adversely affect the strength and corrosion resistance [6]. There are different methods for the welding of metals, such as friction, ultrasonic, laser and spark plasma welding (SPW) [[7], [8], [9], [10], [11]]. Recently, the SPW process has been used for welding dissimilar and similar metals at low temperatures within a short time [[12], [13], [14], [15], [16]]. The most important advantage of the SPW process is the possibility of controlling the microscopic structure and better grain size due to the lower temperature and shorter time of this process, as compared to frictional welding [17]. Extensive research has been performed on the welding of titanium and alloys with other dissimilar materials. In this regard, Miriyev et al. [18] successfully welded the Ti–6Al–4V alloy to steel 4330. Daihua et al. [19] also carried out the welding of Ti-6-Al-4V alloy via the SPW process. They found that the tensile strength of the joint was 78% of that of the substrate matrix. Their results also showed that despite the development of the TiC intermetal layer, the tensile strength of the joint was 250 MPa. Ananthakumar et al. [20] also performed titanium-to-steel welding via the SPW process. By investigating the joint at different temperatures, the joint created at 650 °C showed the best mechanical properties, as compared to other temperatures; the compressive strength and microhardness were obtained to be 429 MPa and 290 Vickers, respectively.

In the present study, for the first time, the diffusion bonding of C·P titanium to Ti-6-Al-4V via the SPW process has been investigated; the effect of temperature, pressure, and time of SPW process on the mechanical properties of the joint was then examined.

## 2 Experimental

In this section, materials, welding methods and characterization methods are discussed.

### 2.1 SPW-assisted diffusion bonding process

**Fig. 1:**
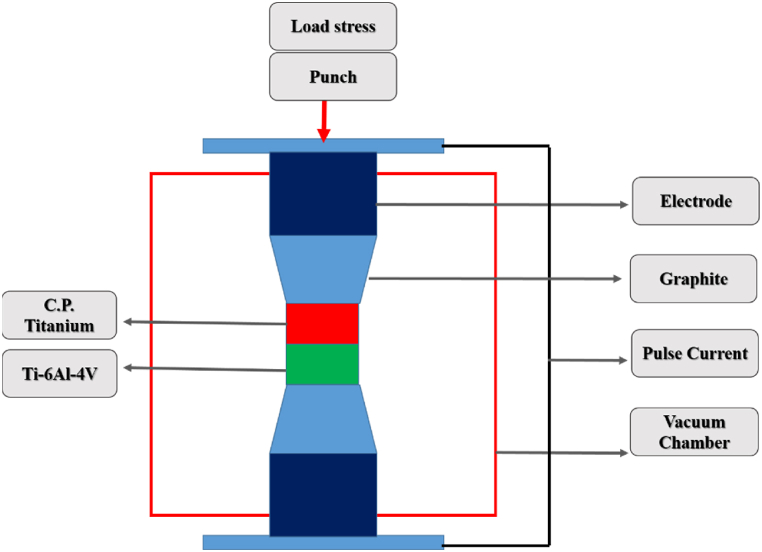
The schematic of SPW.

The starting materials used in this study were commercial pure titanium (C·P) rod (99.98% pure, ILIA S. A. Co, Iran) and Ti–6Al–4V alloy (ILIA S. A. Co, Iran). The chemical composition of both alloys is shown in Table 1. The samples were cut by a wire cut device 20 mm in diameter and 15 mm in height. Before joining, the surfaces of the samples were mechanically polished to ensure ﬂat and smooth surfaces. The samples were then pickled with an acid solution having the composition of 25% HNO_3_ (purity 99.9%) + 5% HF (purity 99.9%) + 70% H_2_O (DI) (vol%) (Merck Co.) for 5 min and washed with acetone (purity 99.9% from Merck Co.) prior to joining. Fig. 1 shows the schematic of SPW. The bonding process was carried out by spark plasma welding techniques, using the machine (Model: SPW10A, MUT Uni, Lab SPS, Iran) at the vacuum atmosphere (0.1 torr). Pulse-mode DC current (pulse 12 ms, pause 2 ms) around 7000 A and a very low voltage of 5 V were applied too. Three parameters of temperature, pressure and time of the SPW process were then investigated. Considering the transformation temperature of the current phases and the melting point in both metals, bonding was carried out at different temperatures with the heating rate of 200 °C/min, as shown in Table 2. To investigate the effect of pressure (stress) on the joint, bonding was performed under the stresses of 20, 40 and 60 MPa with a pressure rate of 5 MPa/min. To investigate the effect of the soaking time on the joint, bonding was carried out at three times of 1, 5 and 10 min. After bonding the samples, they were sectioned using a wire cut device, so that metallography would be performed on the joint section and the quantitative properties of the joint could be investigated. Those sections of the samples were polished with the sandpaper 80 and 5000 and then surface finished on broadcloth by a diamond polishing paste. Eventually, the samples were etched in Kroll etchant and Alpha solution.

**Table 1:** Chemical composition of Ti–6Al–4V and C·P Ti

Chemical Composition	V	Al	C	Fe	Sn	Zr	Si	Cr	Ni	Ti
Ti–6Al–4V	4.38	6.48	0.369	0.112	0.0625	0.0028	0.0222	0.0099	0.001	balance
C·P Ti	0.02	0.01	0.01	0.12	–	–	0.03	0.01	0.01	balance

**Table 2:** Diffusion coefficient of Al into Ti at different temperatures.

C) ° Temperatures (	(m^2^/s) D	(m^2^/s) D_0_	Q (KJ/mol)
700	0.73 × 10^−17^	5.31 × 10^−6^	220.8
750	2.7 × 10^−17^	5.31 × 10^−6^	220.8
800	9.02 × 10^−17^	5.31 × 10^−6^	220.8
850	28.14 × 10^−17^	5.31 × 10^−6^	220.8

### 2.2 Performance evaluation and microstructure analysis

To examine the microstructure of the joint interface, scanning electron microscopy images were captured by 450 QUANTA PEG devices. To examine the thickness of the interface and morphology of the structure, scanning electron microscopy (SEM) images were taken by applying the QUANTA PEG 450 device. Also, to inspect the chemical composition of the joint interface, the chemical composition was examined using this device, which was equipped with X-ray energy diffraction spectrometry. The microhardness test was then performed with the load of 100 g, duration 10 s, as a sloped profile from the joint interface. To investigate the tensile strength, the standard sample of the tensile test was prepared according to the ASTM A370 standard and performed three times with a stretching rate of 0.5 mm/min at room temperature.

## 3 Results

In this section, the results of various analyses concerning the characterization and identification of the microstructure and mechanical properties of the joint are discussed.

### 3.1 Effect of applied pressure

In the welding via the SPW process, the pressure would lead to the development of an active metal surface through the fracture of the surface oxide layer, allowing for the creation of the joints between active metal surfaces. Specifically, the pressure causes an increase in the number of sparks, thereby enhancing the diffusion throughout the entire diffusion-bonded interface [21]. The effect of pressure can be examined as an essential parameter in the SPW process. In 2006, Munir et al. [22] modified the effect of pressure as equation (1), according to the findings of Jamnik and Ruj [23]:

(1) $$\mu_{I}=\mu_{i}^{{}^{\circ}}-\sigma_{n}\Omega_{I}$$,

where $\mu_{I}$ represent the chemical potential, $\mu_{i}^{{}^{\circ}}$ indicates the reference and standard chemical potential, $\sigma_{n}$ denotes the normal stress at the joint interface, and $\sigma_{n}$ shows the atomic volume of the diffusing species [23]. The negative sign of normal stress in the formula represents compressive stress. According to the formula, with increasing pressure (normal stress), the chemical potential is diminished. Since the direction of diffusion is always from areas with the higher chemical potential to the lower ones, the elevation of pressure in the SPW process changes the interface to a suitable place for diffusion between the two alloys.

**Fig. 2:**
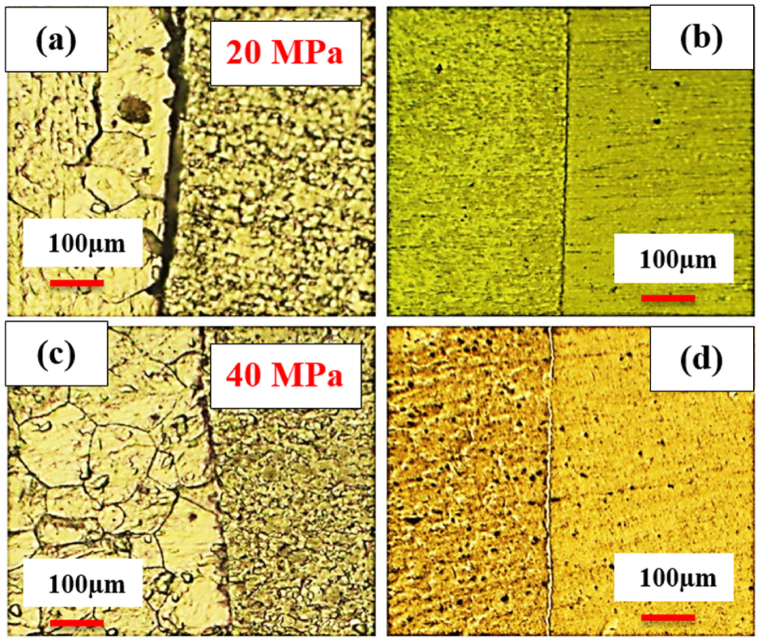
Optical micrographs of cross-sectioned diffusion bonded at different pressure: (a and c) after etching, (b and d) before etching.

**Fig. 3:**
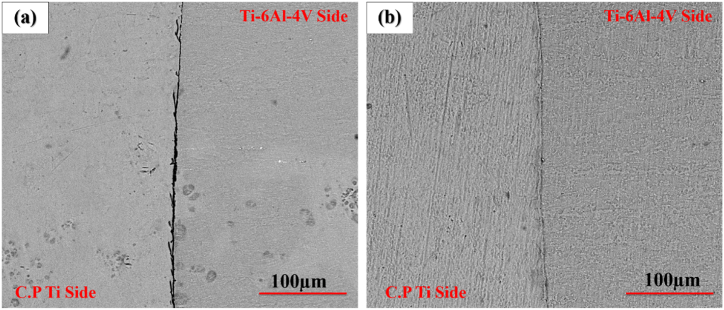
SEM micrographs of cross-sectioned diffusion bonded at different pressure: (a) 20 MPa and (b) 40 MPa.

To examine the effect of pressure, welding was performed under the pressure of 20 MPa and 40 MPa at 750 °C with a retention time of 5 min. Fig. 2(a–d) displays the optical microstructural of diffusion-bonded C·P Ti–Ti64 at 20 MPa and 40 MPa. According to (Fig. 2a and b), at the pressure of 20 MPa, diffusion bonding was not realized completely, and there was a huge gap within the structure. With the elevation of pressure from 20 to 40 MPa (Fig. 2c and d), the gap between C·P Ti and Ti–6Al–4V was eliminated. The increase of stress leads to greater transformation, through which the contact area is enlarged, causing the elimination of the gap between these two structures providing the path required for atomic diffusion. Fig. 3 indicates the SEM microstructure of the diffusion bonding at 20 MPa and 40 MPa. SEM results also confirmed the optical ones. Fig. 3a shows diffusion bonding at the pressure of 20 MPa, showing the gap between the two structures. Fig. 3b also indicates the diffusion bonded at 40 MPa, which was continuous and uniform, with a diffusing zone 2–3 μm thick. The results of the line scan analysis of the diffusion bonded at 40 MPa can be seen in Fig, 4. It was found that Al was diffused from the Ti–6Al–4V side towards Ti, creating a weak concentration gradient at the joint interface in the Ti zone. The element V shows the lower diffusion due to two reasons: i) low solubility of V in Ti with the HCP structure, where this level of solubility is 4%, while the Al solubility in Ti is 25%, and ii) different atomic sizes between V and Ti, which is close to 10%, while this difference between Al and Ti is 2%. Therefore, the diffusion of V into Ti is very low [24].

**Fig. 4:**
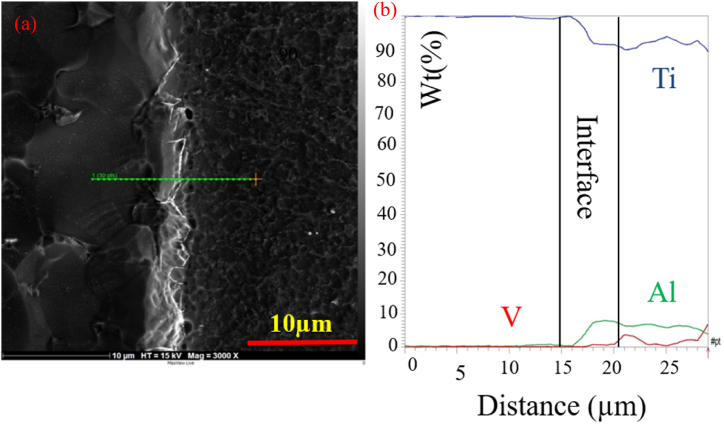
(a) SEM image, BSE mode (b) EDS line scan results of the diffusion bonded C·P Ti–Ti64 at 40 MPa.

### 3.2 Effect of time

Time has double effects on welding via the SPW process. With the excessive prolongation of time in welding via the SPW process, grain growth occurs and mechanical properties are diminished. Meanwhile, if the duration is too short, because of the insufficient opportunity of atoms for diffusion, the bonds are formed incompletely. To better understand the effect of time on the diffusion and eventual development of a suitable joint, by using the Gauss error function [25], the time required for diffusion at 750 °C was calculated against the amount of diffusion within 10 min at 800 °C (equation (2)):

(2) $$\mathrm{erf}\left(Z\right)=\frac{2}{\sqrt{\pi }}\int_{0}^{z}e^{{-y}^{2}}\text{dy},z=\frac{x}{2\sqrt{\text{Dt}}}$$

For maximum values up to 0.5, the Gauss error function can be approximately written as follow (equation (3)):

(3) $$\frac{C_{S}-C_{X}}{C_{S}-C_{0}}≅\frac{X}{2\sqrt{\text{Dt}}}$$

with this in mind, under similar diffusion conditions, the following relation will also hold according equation (4):

(4) $$\frac{{X_{A}}^{2}}{D_{A}t_{A}}=\frac{{X_{B}}^{2}}{D_{B}t_{B}}$$,

where ${X_{A}}^{2}$ and ${X_{B}}^{2}$ represent the distance of the diffusion of the elements A and B, respectively. Also, D_1_ and D_2_ indicate the diffusion coefficient of elements A and B, and t_1_ and t_2_ represent the time required for the diffusion of the elements Al and B in long x [25]. According to formula 5, at 800 ^°^C, Al diffusion coefficient in titanium is $9.02\times 10^{-17}\;m^{2}/s$, which is diffused as long as X for 10 min. Meanwhile, the Al diffusion coefficient in titanium at 750 ^°^C is $2.7\times 10^{-17}\;m^{2}/s$. The diffusion distance at both temperatures is considered constant. By replacing the values in formula 4, the time required for the Al diffusion into Ti at 750 °C, as long as X, can be obtained. This value was achieved in 32 min under these conditions. This means that if the diffusion bonded is established at 750 ^°^C, to achieve the amount of diffusion bonded at 800 °C, the welding time should be 32 min at 750 ^°^C, so that a diffusion bonding similar to the one established at 800 ^°^C would be obtained. These results, thus, indicate that the effect of time cannot be the main influential factor on welding via the SPW process.

**Fig. 5:**
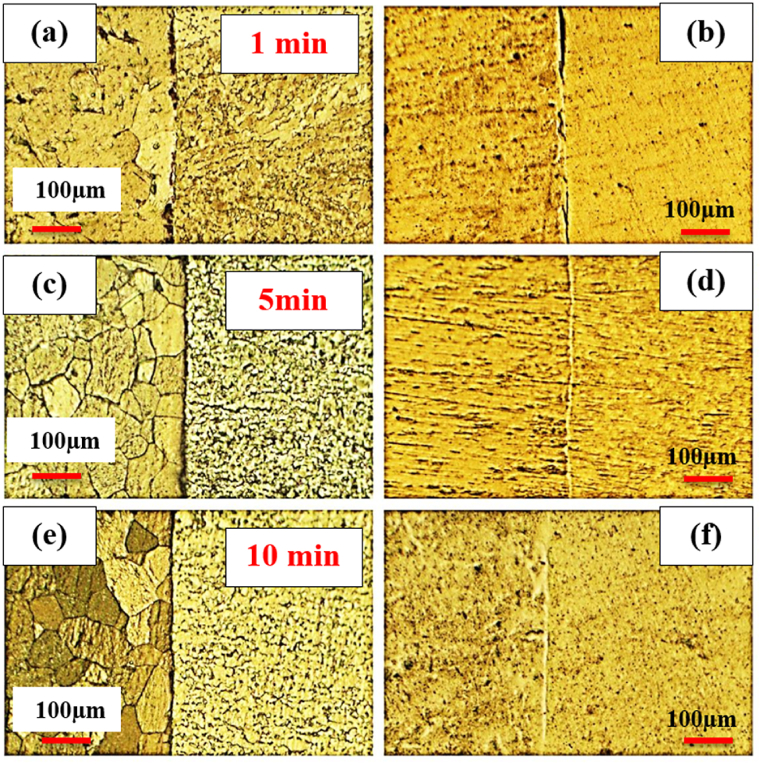
Optical micrographs of cross-sectioned diffusion bonded obtained at a different time: (a, c, e) after etching, (b, d, f) before etching.

**Fig. 6:**
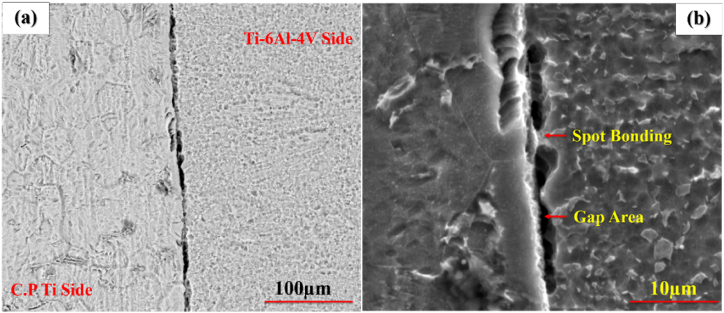
SEM micrographs of cross-sectioned diffusion bonded at 1 min: (a) backscattered electron mode, (b) secondary electron mode.

**Fig. 7:**
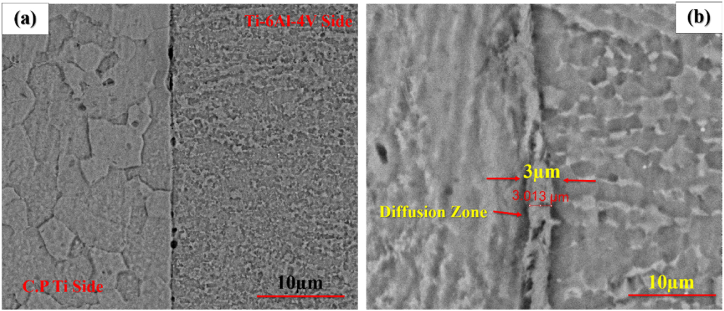
SEM micrographs of cross-sectioned diffusion bonded at 5 min: (a and b) backscattered electron (BSE) mode with magnification 800X and 3000 X respectively.

**Fig. 8:**
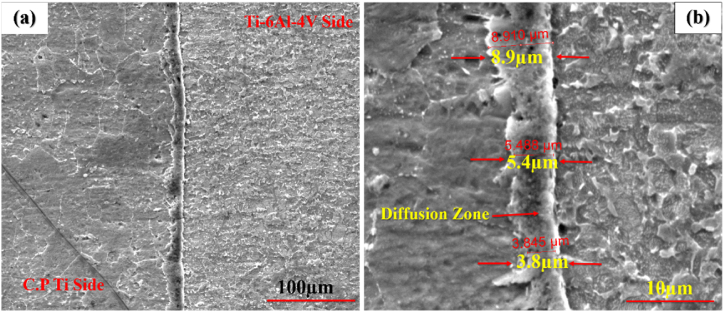
SEM micrographs of cross-sectioned diffusion bonded at 10 min: (a and b) secondary electron mode with magnification 800X and 6000 X respectively.

**Fig. 9:**
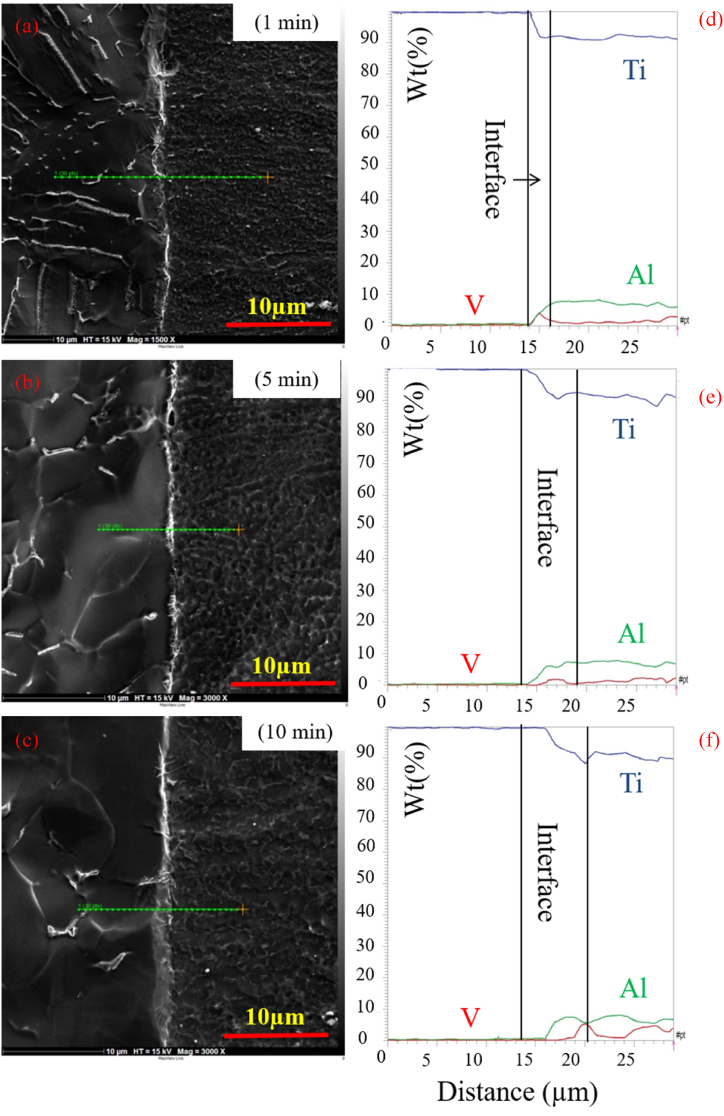
(a–c) SEM image (d–f) EDS line scan analysis of the diffusion bonded C·P Ti–Ti64 at three different times.

**Fig. 10:**
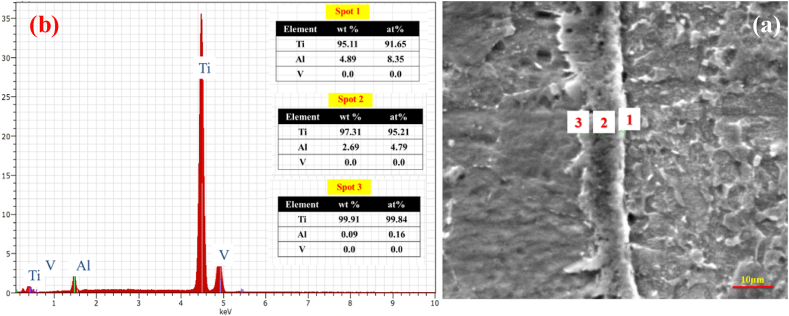
(a) SEM image (b) EDS spot analysis of the elements across the interface between diffusion bonded C·P Ti–Ti64 at 800 ^°^C, 20 MPa, and 10 min.

To explore the effect of time, welding was performed at 1, 5, and 10 min at 800 ^°^C and 20 MPa. Fig. 5(a–f) indicates the optical microstructural of diffusion-bonded Ti–Ti64 at different times. As can be seen, with an increase in the welding time from 1min (Fig. 5a and b) to 5 min (Fig. 5c and d), the gap between the Ti–6Al–4V and C·P Ti was diminished. With the time of 5 min (Fig. 5 c and d) and 10 min (Fig. 5 e,f), in addition to the joint, the diffusion region was also formed on the titanium side. The reason for the formation of the interface on the titanium region side could be the lower melting temperature of C·P Ti, as compared to Ti-6-Al-4V. This caused the interface to move towards the metal with a lower melting point, which was along the direction of the movement of metal atoms with a higher melting point [26]. Fig. 6, Fig. 7, Fig. 8 indicate the SEM microstructure of the diffusion bonding at different times (1, 5, 10 min). According to Fig. 6 (a,b), there was some gap at the diffusion-bonded interface; however, in parts of the joint, a metallurgical bond resulting from diffusion was observed. With the increase in the time of welding, the diffusion area began to form; with increasing the time to 5 min (Fig. 7a and 7b)) and 10 min (Fig. 8a,b), this region was formed with the thicknesses of 2–3 and 5–8 μm, respectively. Fig. 9(a–f) represents the results of line scan analysis at three different times. At 1 min (Fig. 9a–d), it was found that the concentration gradient of Al from Ti–6Al–4V was not diffused towards the Ti side, but this concentration gradient was diffused into the Ti region at the diffusion-bonded interface with the time of 5 (Fig. 9b–e) and 10 min (Fig. 9c–f). Fig. 10 indicates the EDAX spot results from different points of the diffusion region (Fig. 10a) of the joint at 800 °C, 20 MPa, and 10 min (Fig. 10b). By moving further away from the interface of diffusion bonding to the end part of the diffusion region, the amount of Al was diminished.

### 3.3 Effect of temperature

Temperature is an important variable influential on the strength of diffusion bonding via the SPW process. Formation of metal-metal bonds by applying pressure occurs through plastic flow, creep deformation, and diffusion along surfaces and grain boundaries or the volume of one grain. Elevation of temperature causes the increase of the contact area between the materials by reducing the yield strength and increasing the diffusion rate; it also controls the removal of pores, growth of grains, and chemical reactions [27]. Thus, the diffusion bond would be created within a shorter time. For a more precise investigation of the effect of temperature on atomic diffusion, by considering the Arrhenius formula (1), aluminum into titanium diffusion coefficient at different temperatures of welding was calculated (equation (5)) [26,28,29]:

(5) $$D=D_{0}\;\exp\;\left(-\frac{Q}{\text{RT}}\;\right)$$

In this relation, D is the diffusion coefficient, D_0_ represents the frequency factor, Q denotes the activation energy for diffusion, R is the constant of gases, and T is the temperature (K). The frequency factor and activation energy for aluminum into titanium diffusion for a compound containing Al 6 wt% is D_0_ = 5.31 × 10^−6^ [30] and 220.8 kJ/mol [30], respectively. Assuming D_0_ and Q constant, the diffusion coefficient was calculated at 700, 750, 800, and 850 °C, as shown in Table 2. According to formula 5, with the elevation of temperature from 700 to 850 °C, the diffusion coefficient was increased from D_0_ = 0.73 × 10^−17^ to D_0_ = 28.14 × 10^−17^. This means that with only a 150 ^°^C increase, the diffusion coefficient was grown by around 27 times. These results, thus, suggested that the diffusion rate heavily grew with temperature elevation, through which the welding time would be decreased.

**Fig. 11:**
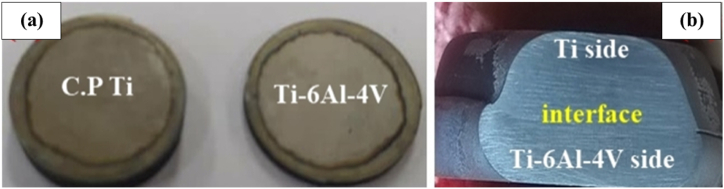
Photographs of C·P Ti–Ti64 diffusion bonded at: (a) 700 ^°^C and (b) 850^°^C.

To explore the effect of temperature, welding was performed at 700, 750, 800, and 850 °C, with a pressure of 20 MPa, and a retention time of 5 min. The images of the samples welded at 700 and 850 °C are represented in Fig. 11. According to image Fig. 11a, no joint was created at 700 C. At this temperature, because of the low diffusion coefficient, atomic diffusion and diffusing pair formation did not occur. Meanwhile, Fig. 11b indicates the joint at 850 °C; because of the high diffusion coefficient and very high plastic transformation at this temperature, the sample was changed into a full barrel-shaped pill-like form. By comparing the results of Fig. 1 (a,b) and Fig. 4 (c,d), as well as the SEM results of Figs. 2 (a) and Fig. 6 (a,b), which represent the diffusion bonded at 750 and 800 °C, it was found that as the temperature was increased, the diffusion coefficient was raised too; as a result, the gap between the two structures was eliminated through temperature elevation, and the diffusion region was also formed.

## 4 Evaluation of mechanical properties

**Fig. 12:**
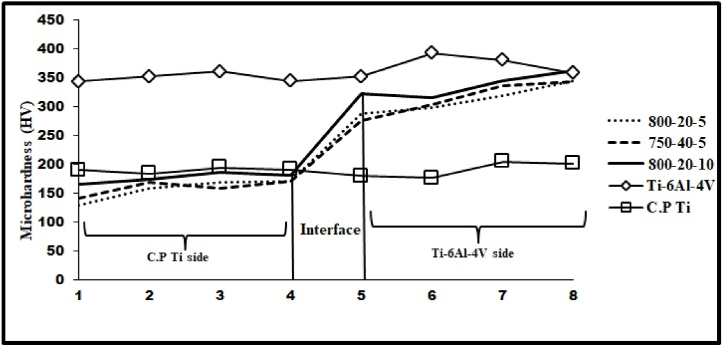
Micro hardness value along the interface region at different SPW processes.

**Fig. 13:**
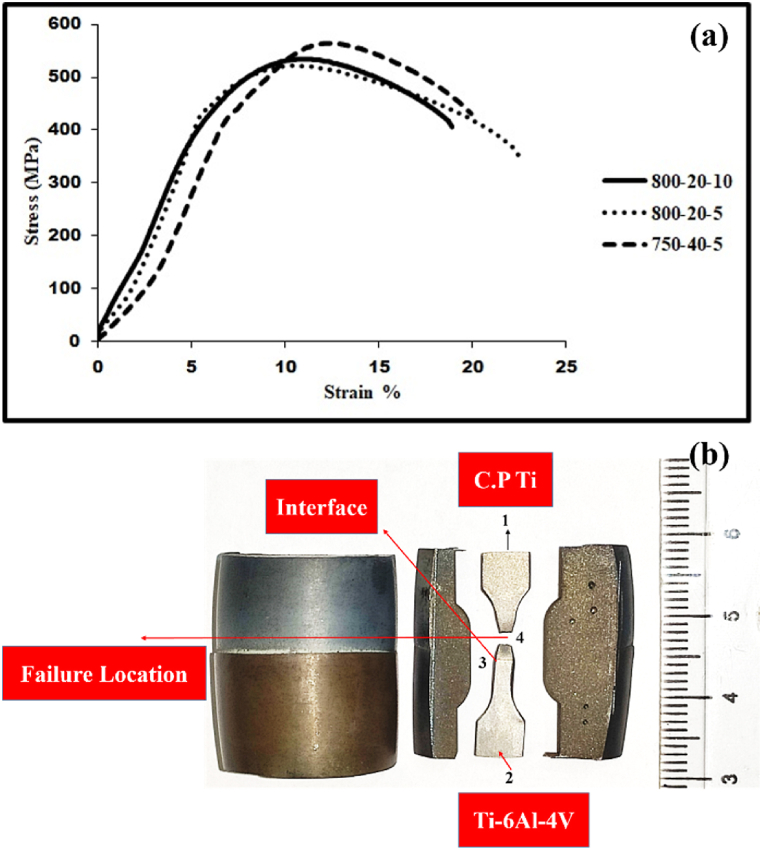
(a) The stress-strain diagram and (b) C·P titanium/Ti–6Al–4V tensile sample joint by SPW.

The stress-strain diagram and the C·P titanium/Ti–6Al–4V tensile sample joint developed by applying spark plasma welding are represented in Fig. 13 (a,b). The tensile test was performed for all samples; only in the three samples presented in Table 3, the strength of the joint interface was higher than that of the substrate metal. The results obtained from the tensile test, thus, showed that in samples b, c, and e, necking occurred from the titanium side, and eventually fracture started from the titanium side. The higher strength of the joint interface, as compared to the titanium substrate metal, suggested that a full metallurgical bond had been established between Ti–6Al–4V and C·P Ti, which was in line with other results. The largest UTS value was related to the sample (c), which had been welded within a shorter time, as compared to the sample (b). This could be attributed to the greater grain growth in the sample (b), as it had been retained at the temperature twice longer than that done in the sample (c). This grain growth was followed by a decline in strength and an increase in flexibility. Fig. 12 displays the diagram of the microhardness of the joint of samples b, c, and g alongside Ti–6Al–4V and C·P Ti. It was found that the hardness of Ti–6Al–4V and C·P Ti did not change considerably after the SPW process. However, in the some-micron region close to the interface in the Ti–6Al–4V area, the hardness was lower, while in the C·P Ti area, the hardness was higher. The mechanical properties produced by the SPW method were compared with those obtained by other methods, as shown in Table 4. The results showed that the mechanical properties of C·P Ti joined to Ti–6Al–4V by the SPW method were more acceptable than other methods. The reason could be the lack of change in the microstructure of dissimilar metals during joining. Therefore, no intermetallic compounds were formed.

**Table 3:** Results of tensile test on C·P Ti–Ti64 diffusion bonded at different SPW process.

Sample	(°C) Temperature	(MPa) Pressure	(min) Time	UTS (MPa)	Elongation (%)	Fracture
A	800	20	1	320	3	Interface
B	800	20	5	564	11	Ti
C	800	20	10	534	15	Ti
D	750	20	5	119	1	Interface
E	750	40	5	522	12	Ti

**Table 4:** Comparison of the mechanical properties of this research with other studies.

Type of Joint	Joint method	Tensile Strength (MPa)	micro-hardness (HV)	Fracture Location	References
C·P Ti/Ti64	FLW	345	290	C.P-Ti side	[26]
G.2 Ti/Ti64	LBW	–	300	joint Interface	[27]
G.2 Ti/Ti64	LBW	433	400	joint Interface	[28]
C·P Ti/Ti64	Brazing	261	450	joint Interface	[29]
Ti/304L	SPW	250	510	joint Interface	[15]
Ti/304L	SPW	263	–	joint Interface	[13]
Ti/304L	SPW	210	350	joint Interface	[30]
Ti/304L	SPW	320	390	joint Interface	[31]
Ti64/Ti64	SPW	886	–	Base side	[14]
C·P Ti/Ti64	SPW	534	190	C·P Ti	This Research

## 5 Principle of spark plasma welding

In diffusion welding via the SPW method, the applied transformation first leads to the breakdown and loss of rough surfaces because of the applied pressure. Since the initial contact area is small, this stage results in better and greater welding, thus facilitating the welding further for the subsequent stages. At this stage, some networks of closed pores are created in the interface [22].

The transformed material across the connection may be the metal itself or an intermediate layer placed between two heterogeneous materials. Pressure causes the breakdown of the oxide layer of the metal surface and creates an active metal surface. This causes the direct contact between the surface of active material (A) and the other material (B). Considering the type of heterogeneous samples, different reactions may cause the development of the connection. For example, in the Al–Mg and zirconia connection, Al and Mg atoms react with the grain boundary phases of zirconia and zirconia itself (ZrO_2_), leading to the development of the connection [[16], [17], [18], [19], [20], [21], [22], [23], [24], [25], [26], [27], [28], [29], [30], [31], [32], [33], [34], [35], [36], [37], [38]].

As stated earlier, diffusion welding requires plastic deformation and yielding; so at least one of the components would reduce the remaining pores at the interface, leading to the development of the suitable connection. This continues until we reach the nominal area of the connection, where the pores have been removed completely. When the main connection area is developed and the bones are established at the connection interface, local stresses decrease inversely in relation to the actual area of the connection. The plastic flux rate at the connection region is determined by the level of local stresses at the welding temperature, and it decreases with the enlargement of the actual connection area. Although local stresses decrease with the enlargement of the connection area, the major time of connection is related to the last stage, which is associated with the removal of the separate pores. At this final stage, the radius of curvature of separate pores creates a driving force for the closure of course. We will have two types of remaining pores: those that are larger and result from the geometric mismatch at mesoscopic size, and the smaller ones which are microscopic and related to the initial surface topology. The microscopic pores are much harder to eliminate, but the mesoscopic ones can be dwindled easily. This is performed through rougher abrasion in the last stage of surface abrasion; so the distance between the main points of the connection would decrease, thereby preventing the formation of large and separate pores. The optimal results are often obtained for special roughness (the size of the abrasive material). Nevertheless, there are still tiny pores that can accumulate under the hydrostatic capillary pressure. Thus, in the welding terminal stage, when the capillary pressure increases above the applied pressure, the initial surface area can be a better variable, as compared to the initial welding bond. Another important issue in diffusion welding is the migration and diffusion of atoms in the interface. Contraction of pores depends on the rate of the movement of materials in the solid phase, as well as the solid motion mechanism, which is determined based on the activation energy of the process. Surface diffusion, boundary diffusion, and volume diffusion are the three possible mechanisms. Boundary diffusion, which is the most common state of diffusion, involves the movement of the voids of the network from the pore surface to the boundary, i.e. where they are absorbed. Thus, the contraction of pores is associated with the migration from the center of the gravity of the grains towards the boundary plane [[37], [38], [39], [40]].

## 6 Conclusion

SPW is a promising method for joining materials. The three parameters of temperature, time and pressure in the SPW method play an essential role in creating an acceptable joint. In the case of joining C·P Ti to Ti–6Al–4V alloys, it was shown that:

1. C.P Ti to Ti–6Al–4V joining was processed using the SPW technique in the 700–850 °C temperature range for 1–10 min under the pressure of 20–60 MPa.
2. SPW technique is an effective approach for joining C·P Ti to Ti–6Al–4V, with the tensile strength and hardness of the joints being about 534 MPa and 190 HV, respectively.
3. A thin (∼9 μm) diffusion zone layer formed in the joined region separates the joined metals and prevents the formation of the intermetallic compounds.
4. Analysis of the temperature effect indicated that with the rise of temperature from 750°C to 800 °C, because of the excessive heating of the contact area between the two metals and the increased diffusion coefficient, a stronger joint would be established between the two metals through the metallurgical bond.
5. Investigation of the effect of time showed that with the prolongation of time from 1 to 10 min, the amount of gap between the two structures was diminished; eventually, the diffusion area began to form; so, the longer the time, the greater the thickness of this diffusion area.
6. Investigation of the effect of pressure (normal stress) also indicated that with the elevation of pressuredue to the reduction of the chemical potential of the diffusing species, the welding temperature was diminished, and the joint could be created at a temperature below 800°C.
7. No welding of large parts, the creation of intermetallic compounds and the possibility of changing the microstructure in conventional welding methods such as FLW, LBW and FW for joining the C·P Ti alloy to the Ti–6Al–4V alloy could lead to the emergence of solid state joining methods. The joining process in a very short time in the SPW method leads to joining dissimilar materials with the maximum mechanical properties.

## Data availability

Data will be made available on request.

## CRediT authorship contribution statement

**Saeed Zahabi:** Writing – original draft, Methodology, Investigation, Formal analysis, Data curation, Conceptualization. **Ehsan Mohammad Sharifi:** Writing – review & editing, Supervision. **Mehdi Naderi:** Visualization, Validation, Project administration, Methodology, Investigation. **Mazaher Ramezani:** Supervision, Project administration, Methodology, Funding acquisition, Formal analysis. **Hossein Jamali:** Writing – review & editing, Validation. **Mohammad Reza Loghman Esatrki:** Writing – review & editing.

## Declaration of competing interest

All authors state that there are no conflict interests.
